# Ten Nearly Complete Genome Sequences of Human Orthorubulavirus 4 Isolated from Pediatric Inpatients in Fukushima, Japan

**DOI:** 10.1128/mra.00411-22

**Published:** 2022-06-09

**Authors:** Satoko Sugimoto, Yohei Kume, Reiko Suwa, Miyuki Kawase, Masatoshi Kakizaki, Kazutaka Egawa, Takashi Ono, Mina Chishiki, Hisao Okabe, Sakurako Norito, Masatoki Sato, Hiroko Sakuma, Shigeo Suzuki, Mitsuaki Hosoya, Makoto Takeda, Koichi Hashimoto, Kazuya Shirato

**Affiliations:** a Department of Virology III, Laboratory Animals, and Pathogen Bank, National Institute of Infectious Disease, Musashimurayama, Tokyo, Japan; b Management Department of Biosafety, Laboratory Animals, and Pathogen Bank, National Institute of Infectious Disease, Musashimurayama, Tokyo, Japan; c Department of Pediatrics, School of Medicine, Fukushima Medical University, Fukushima, Japan; d Hoshi General Hospital, Koriyama, Fukushima 963-8501, Japan; e Ohara General Hospital, Fukushima, Japan; Queens College CUNY

## Abstract

We report 10 nearly complete genomic sequences of human orthorubulavirus 4, also called human parainfluenza virus 4 (HPIV4), isolated from pediatric inpatients with respiratory infections in Fukushima, Japan, by using an air-liquid interface culture of human bronchial and tracheal epithelial cells.

## ANNOUNCEMENT

Human parainfluenza viruses (HPIV) are members of the family *Paramyxoviridae* with genomes consisting of approximately 15 to 17 kb of single-stranded negative-sense RNA (1, 2). There are four HPIVs: HPIV1 and HPIV3 belong to the genus *Respirovirus*, whereas HPIV2 and HPIV4 belong to the genus *Orthorubulavirus*. HPIV4 is subdivided into two groups (HPIV4a and HPIV4b) based on antigenicity (3). HPIV4 was first identified in 1959 (4); despite more than 60 years passing since its identification, understanding of HPIV4 still remains limited owing to the difficulty of isolating this virus (5), and only a few HPIV4 genomic sequences are registered in GenBank. The air-liquid interface culture of human bronchial and tracheal epithelial cells (HBTEC-ALI) is an excellent tool for culturing various respiratory viruses, particularly those that are hard to isolate, such as human coronaviruses (6, 7), human bocavirus (8), and rhinovirus (9). Here, 10 nearly complete genome sequences of HPIV isolates were determined (Table 1). Nasopharyngeal swabs were collected from pediatric inpatients in Fukushima, Japan from 2018 to 2022, and those that were identified as HPIV4-positive by real-time PCR for respiratory viruses (10, 11) were used for virus isolation. HBTEC-ALI-cultured cells were prepared as described previously (6, 12). At 7 days after inoculation onto HBTEC-ALI culture with specimens, cells were washed with culture medium, and the presence of virus in cell wash was confirmed by real-time reverse transcription-PCR. Cell wash that was HPIV4-positive was stored as virus stock. Finally, 10 isolates were obtained from three mono-infections and seven coinfections with another respiratory virus (Table 1). Nucleic acids were extracted from virus stock by using the ISOGEN-LS reagent (Nippongene, Tokyo, Japan) following the manufacturer’s protocol. The libraries for next-generation sequencing were prepared using a NEBNext Ultra II RNA library prep kit for Illumina (New England Biolabs, Ipswich, MA, USA) following the manufacturer’s instructions. The indexed libraries were analyzed for 2 × 150 cycles on a DNBSEQ-G400 instrument at GENEWIZ (South Plainfield, NJ, USA) or on a Miseq with reagent kit v3 (Illumina, San Diego, CA, USA) at our institute. Reads were trimmed and then *de novo* assembled using CLC Genomics Workbench v21.0.4 on the default settings. The average coverage was checked by mapping reads to assembled sequence, and assembled sequences were trimmed by comparison with the reference sequences (e.g., GenBank accession numbers MN306056 for HPIV4a and KY986647 for HPIV4b).

**TABLE 1:** List of registered HPIV4 sequences

HPIV sequence name	Accession no.	Run data accession no.	Total reads	Total mapped reads	Avg coverage	Length (bases)	GC content (%)	Coinfection^*a*^
PIV4a_Fukushima_O113_2018	LC706548	DRR360803	245,016,168	1,732,756	14304.77	17,094	36.22	Mono
PIV4a_Fukushima_H407_2018	LC706552	DRR360804	570,524,308	4,008,643	33,298.44	17,094	36.22	RSV B
PIV4a_Fukushima_H725_2019	LC706553	DRR360805	320,784,375	2,284,465	18,731.99	17,094	36.19	ADV2
PIV4a_Fukushima_O521_2019	LC706549	DRR360806	440,731,019	3,150,837	25,721.55	17,100	36.29	Mono
PIV4a_Fukushima_O660_2019	LC706550	DRR360807	14,376,859	100,222	840.4	17,094	36.19	PIV3
PIV4a_Fukushima_O755_2019	LC706551	DRR360808	82,215,982	567,578	4,802.16	17,094	36.19	RSV B
PIV4b_Fukushima_O896_2019	LC706554	DRR360809	3,731,778	25,831	214.5	17,384	36.39	ADV2
PIV4b_Fukushima_OR473_2022	LC706555	DRR360810	2,973,974	39,183	170.24	17,359	36.31	HPIV2
PIV4b_Fukushima_OR476_2022	LC706556	DRR360811	46,013,446	606,106	2649.26	17,308	36.27	Mono
PIV4b_Fukushima_OR487_2022	LC706557	DRR360812	1,688,111	22,232	97.04	17,361	36.28	ADV2

a Coinfection was determined by multiplex real-time PCR assays for respiratory viruses (10, 11). RSV, respiratory syncytial virus; ADV, adenovirus.

Using these methods, nearly complete genome sequences of six HPIV4a and four HPIV4b isolates were determined (Table 1). With these 10 sequences, the total number of registered HPIV4 genome sequences has increased greatly, although it is still less than 40. HPIV4 can be isolated from pediatric inpatients with respiratory infections, indicating its importance in pediatric infections. We expect that more viruses will be isolated using HBTEC-ALI culture, and their genomic sequences will be decoded to evaluate the genetic evolution of HPIV4.

### Data availability.

The nearly complete genome sequences have been deposited in GenBank under the following accession numbers (Table 1): LC706548, LC706549, LC706550, LC706551, LC706552, LC706553, LC706554, LC706555, LC706556, and LC706557. The raw reads were deposited under BioProject number PRJDB13434. Each run data sequence has been deposited in the DNA Data Bank of Japan under the following accession numbers: DRR360803, DRR360804, DRR360805, DRR360806, DRR360807, DRR360808, DRR360809, DRR360810, DRR360811, and DRR360812.
